# Management of a Girl With Delayed Puberty and Elevated Gonadotropins

**DOI:** 10.1210/jendso/bvac108

**Published:** 2022-07-08

**Authors:** Sinéad M McGlacken-Byrne, John C Achermann, Gerard S Conway

**Affiliations:** Institute for Women’s Health, University College London, London WC1E 6AU, UK; Genetics and Genomic Medicine, UCL Great Ormond Street Institute of Child Health, University College London, London WC1N 1EH, UK; Department of Paediatric Endocrinology, Great Ormond Street Hospital, London WC1N 3JH, UK; Genetics and Genomic Medicine, UCL Great Ormond Street Institute of Child Health, University College London, London WC1N 1EH, UK; Institute for Women’s Health, University College London, London WC1E 6AU, UK

**Keywords:** Turner syndrome, primary ovarian insufficiency, 46, XY DSD, ovary development, primary amenorrhea, delayed puberty, estrogen, support groups

## Abstract

A girl presenting with delayed puberty and elevated gonadotropins may have a range of conditions such as Turner syndrome (TS), primary ovarian insufficiency (POI), and 46,XY disorders of sexual development (DSD). An organized and measured approach to investigation can help reach a timely diagnosis. Management of young people often requires specialist multidisciplinary input to address the endocrine and nonendocrine features of these complex conditions, as well as the psychological challenges posed by their diagnosis. Next-generation sequencing within the research setting has revealed several genetic causes of POI and 46,XY DSD, which may further facilitate an individualized approach to care of these young people in the future. Pubertal induction is required in many and the timing of this may need to be balanced with other issues specific to the condition (eg, allowing time for information-sharing in 46,XY DSD, optimizing growth in TS). Shared decision-making and sign-posting to relevant support groups from the outset can help empower young people and their families to manage these conditions. We describe 3 clinical vignettes of girls presenting with delayed puberty and hypergonadotropic amenorrhea and discuss their clinical management in the context of current literature and guidelines.

Girls with delayed puberty and absent periods can present to a wide range of health professionals. Endocrinologists frequently play a central role in establishing an appropriate diagnosis and management plan. Primary amenorrhea is defined as an absence of periods after age 15 years in girls with a degree of breast development or after age 13 years in those with complete absence of pubertal signs. Delayed puberty is often self-limiting, but occasionally it is the presenting feature of a wide range of underlying conditions, many of which have lifelong management implications. With this in mind, engaging the young person in a sensitive manner and gaining their confidence from the outset is extremely important.

The initial consultation with the young person and appropriate family members needs to include a careful history to establish key features of the presentation and any past medical and family history that may provide insight into an underlying diagnosis. Any examination should be performed sensitively, with the young person’s consent and understanding of why it is necessary and with a chaperone present as appropriate. Assessment should be made of weight and height in relation to mid-parental height and pubertal stage, pubertal development using Tanner stages, the presence of any potentially relevant associated features such as virilization, and blood pressure. Dignity should be maintained and invasive examination avoided. Virilizing conditions are rare and can be assessed by external genital examination for clitoromegaly if suspected. If an intimate examination is required, then it is not essential that this take place on the first visit and it can be postponed until a relationship with the clinical team has developed [1].

For this review, we focus on the girl with *absent (delayed) puberty* and *elevated gonadotropins* (hypergonadotropic amenorrhea), and use 3 case scenarios to highlight key points specific to the individual diagnoses, followed by a discussion on management considerations that are related to conditions presenting with hypergonadotropic hypogonadism. Fig. 1 illustrates how this scenario fits in with other causes of primary amenorrhea.

**Figure 1.**
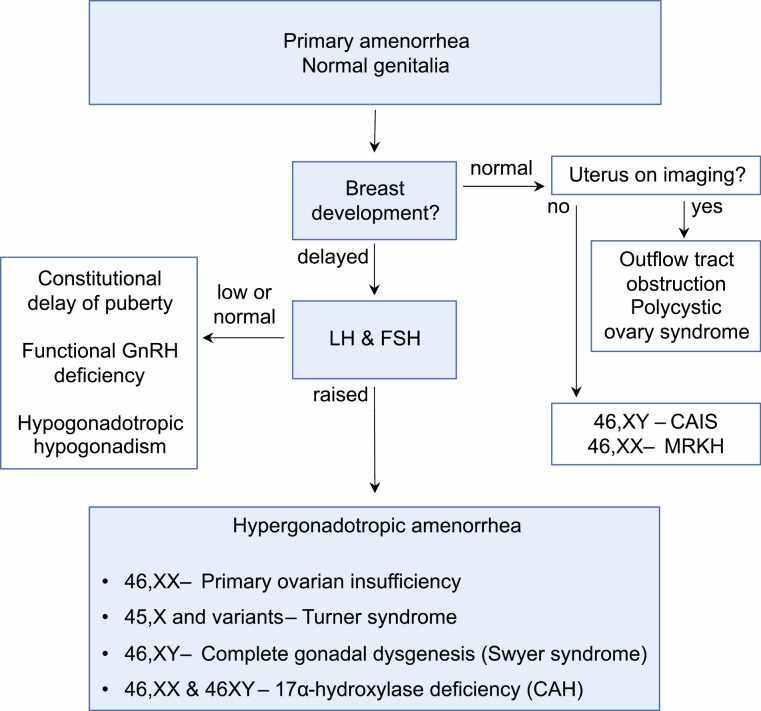
Simplified flow diagram of the pathway of diagnosis of hypergonadotropic amenorrhea and related conditions presenting with primary amenorrhea. CAH, congenital adrenal hyperplasia; CAIS, complete androgen insensitivity syndrome; MRKH, Mayer-Rokitansky-Küster-Hauser syndrome.

## Case 1—Turner Syndrome

A 14-year-old girl presented with short stature, primary amenorrhea, and delayed puberty. Her height was on the third percentile (mid-parental height 75th percentile) and weight on the 10th percentile. Pubertal staging demonstrated Tanner breast stage 2 with no signs of virilization. There was a history of early feeding difficulties and otitis media up to age 5 but no current reported hearing loss. Parents were nonconsanguineous and there were no difficulties at school, although she was feeling increasingly isolated from friendship groups because of her delayed puberty.

Investigations showed normal thyroid function, elevated luteinizing hormone (LH) 25 IU/L and follicle-stimulating hormone (FSH) 61 IU/L, and low antimullerian hormone (AMH) 0.5 pmol/L. A karyotype showed 45,X, consistent with Turner syndrome (TS). Transabdominal ultrasound revealed a small, prepubertal uterus and very small, 0.2-cm [3] ovaries bilaterally with no follicle activity. Bone age was delayed (11 years 8 months). Subsequent investigations included an echocardiogram that showed a tricuspid aortic valve and normal dimensions of the ascending aorta. A renal ultrasound was normal. Other recommended screening tests were unremarkable other than a formal hearing test that revealed mild conductive hearing loss. Treatment was started with growth hormone and titrated up to achieve an insulin-like growth factor 1 within the high-normal range. Pubertal induction was commenced using one-fourth of a 25 mcg 17β-estradiol patch changed twice weekly. Contact was made with a TS support group as well as psychology services within the endocrine multidisciplinary team. Fertility expectations with pregnancy using oocyte donation were discussed shortly after diagnosis.

TS affects 1 in 2500 girls and women and results from complete or partial loss of 1 X chromosome. Karyotypes associated with TS include monosomy X (45,X), 45,X mosaicism (45,X/46,XX, mosaicism with Triple X, or 45,X/46XY), isochromosome Xq, and ring X chromosome. The phenotype of TS is variable and can present at different stages in life but classically TS is characterized by primary ovarian insufficiency (POI) and extragonadal features, including short stature, hypothyroidism, and cardiac defects such as coarctation of the aorta. POI in TS tends to be early onset (50%-85%), presenting with delayed puberty, primary amenorrhea, and hypergonadotropic hypogonadism. TS is thought to account for 10% of females presenting with primary amenorrhea. However, the reproductive phenotype of TS can vary, with approximately 30% of girls with TS having spontaneous thelarche, 15% presenting with secondary amenorrhea, and a further 3% to 5% having ongoing spontaneous menstrual cycles and even achieving spontaneous pregnancies [2-4]. The mechanisms underlying this variability are not clear, although X-chromosome dosage likely plays a role: Mosaic TS is associated with an increased rate of spontaneous pubertal development and fertility [5, 6]. Alternative explanations include altered X-inactivation patterns, haploinsufficiency of pseudoautosomal region genes, or genomic variability in key X-chromosome genes modifying the reproductive phenotype of TS [7, 8].

The presence of Y-chromosome material, that is, 45,X mosaicism involving a Y-cell line, on karyotype or fluorescence in situ hybridization analysis represents a particular challenge as it confers a risk of gonadoblastoma in approximately 10% of these individuals [9]. Prophylactic gonadectomy is recommended in all individuals with TS who have detectable Y-chromosome material and intraabdominal gonads [9].

There are clear international consensus guidelines on how best to diagnose and manage TS [9]. These cover genetic testing, growth, puberty, fertility counseling, cardiovascular health, and health surveillance for comorbidities such as hearing impairment, diabetes, and dyslipidemia. This presentation of an adolescent with TS raises several issues. There may be relief or frustration that past medical issues are now explained by the diagnosis of TS. Input from a psychologist may include liaison with the school for educational review and support needs. Specific to this age group is the conflict between promoting growth with growth hormone and ensuring timely pubertal progression with exogeneous estrogen [10]. Estrogen at high doses has the potential to accelerate closure of the long bone epiphyses, compromising growth and raising the question of whether pubertal induction should be delayed for a period to allow growth hormone take effect. Earlier studies found that relatively high doses of estrogen compromised final adult height and advocated for a delay in estrogen introduction [11]. Later trials, however, demonstrated that low-dose estrogen used in tandem with growth hormone at or after age 12 did not have a significant effect on final height [12-14]. Pubertal induction, and specific considerations in girls with TS, are discussed later.

## Case 2—Primary Ovarian Insufficiency

A 14-year-old girl presented to her primary care doctor because of concern that puberty had not yet started. She was otherwise completely well and excelled academically. A maternal aunt had autoimmune hypothyroidism but there was no other relevant family history. Her parents were first cousins and she had 2 brothers who had entered puberty at an expected age. Her mother had her menarche at age 12, had no difficulty conceiving, and was not menopausal at age 50. Pubertal staging revealed no breast development (B1), sparse pubic hair (P2), and no axillary hair (A1). Height and weight were on the 50th percentile with a mid-parental height on the 75^th^ percentile. Examination was otherwise normal. Investigations revealed positive thyroid peroxidase (TPO) antibodies, markedly raised gonadotropins (LH 42 IU/L; FSH 96 IU/L), and an undetectable serum estradiol concentration.

Further testing at a reproductive medicine unit two months later demonstrated similar gonadotropin concentrations, a 46,XX karyotype, weakly positive TPO antibodies, negative adrenal (adrenal cortex 21-hydroxylase) antibodies, normal thyroid function, and a negative fragile X screen. A diagnosis of POI was therefore confirmed. Puberty was gradually induced with 17β-estradiol transdermal patches (see “Pubertal Induction”). This girl was devastated by the diagnosis of POI and developed depression requiring medication. She benefitted from clinical psychology input and from a POI support group. She was frustrated by the lack of explanation for her condition and joined a research study looking at genetic causes of POI. Trio-exome sequencing revealed a pathogenic homozygous variant within *STAG3*, a gene required for normal human meiosis that has been previously associated with POI.

POI arises from an inherent defect within the ovary that results in estrogen deficiency and infertility secondary to depletion of the follicle pool [15, 16]. POI affects 1% of females and rarely can be early onset, presenting in adolescence with primary amenorrhea, early secondary amenorrhea, or delayed/arrested puberty. POI is diagnosed in those presenting before age 40 years with increased FSH (> 25 IU/L) on 2 occasions at least 1 month apart, biochemical estrogen deficiency, and amenorrhea for a least 4 months [17, 18]. AMH measurement and ovarian ultrasound are of limited diagnostic use in POI and are not routinely indicated clinically [17].

Several causes of POI have been identified. Iatrogenic causes, such as chemotherapy, radiotherapy, and surgery, explain up to 30% of POI diagnoses in some clinical populations [19, 20]. This is likely to rise as survivorship of pediatric cancer continues to improve. Autoimmune mechanisms have also been implicated in the pathogenesis of POI in up to 30% of women [21-23], and autoimmune conditions in general are more frequent in women with POI. Common autoimmune associations include hypothyroidism, autoimmune polyglandular syndrome, Addison disease, celiac disease, and pernicious anemia [21]. Antiovarian antibodies can be positive in varying proportions of women with POI (4%-69%), but the role of these antibodies in the pathogenesis of POI is unclear [24]. Current European Society of Human Reproduction and Embryology guidelines suggest that measuring anti-TPO, adrenocortical, and 21-hydroxylase antibodies can be considered at diagnosis if the clinical picture suggests an autoimmune etiology; if positive, these antibodies can be useful markers of autoimmunity in women with POI [17].

There is also a substantial genetic component to the pathogenesis of POI, supported by the high incidence of familial POI (up to 30%) particularly within consanguineous families [25] and the association of POI with several syndromes (Table 1*).* The most common associated clinical features are short stature (TS) and deafness (Perrault syndrome). Taking a careful family history and performing a systematic clinical examination at the time of presentation is important. X-chromosome abnormalities as well as copy number variants of key X-chromosome region genes (*DACH2*, *XPNPEP2*, *POF1B*, *DIAPH2*) are a recognized cause of POI in 2% to 5% of women with this condition. *FMR1* premutations, associated with fragile X, accounts for approximately 3% of women with POI but rarely presents with primary amenorrhea [29]. In recent years, next-generation sequencing approaches within a research context have identified a variant possibly contributing to a POI diagnosis in up to 50% of women within these studies [30-36]. However, the functional evidence for the pathogenicity of these variants varies. Establishing causality is further hindered by small pedigrees and a remarkably heterogeneous genetic architecture; variants in more than 100 genes have been implicated in the pathogenesis of POI with several postulated modes of inheritance, including oligenic and polygenic inheritance patterns. Table 2 outlines genes within which pathogenic variants have been clearly demonstrated to cause POI in humans. Over recent years these variants have highlighted the importance of developmental processes such as oogenesis and meiosis to normal ovarian function. Abnormalities within genetic mechanisms required for meiosis have emerged in recent years as a significant underlying cause of POI [67]. Meiosis is a complex series of events beginning in fetal life, when homologous chromosomes form the synaptonemal complex. *STAG3* encodes for one of the cohesion proteins that stabilize this complex. Meiotic homologous recombination follows, a process for which DNA repair is essential. Accordingly, pathogenic variants in DNA repair genes (eg, *BRCA2*, *MSH4*, *MSH5*, *MCM8*, *MCM9*, *ZSWIM7)* are among those implicated in the pathogenesis of POI (see Table 2).

**Table 1.** Selected syndromic forms of POI with summary of ovarian phenotype [26-28]

Syndromic POI and clinical synopsis including ovarian phenotype	Chromosome anomaly / gene variants	Inheritance pattern
***Turner Syndrome and variants*** *-* Short stature, web neck, renal anomalies, otitis media, bicuspid aortic valve, coarctation, hypothyroidism. NB 45,X/46,XX mosaicism associated with milder phenotype and late presentation.	Karyotype 45,X, 45,X/46,XX or 45,X/46,XY mosaicism, mosaic ring X, isodicentric Xq, and other variants	Sporadic
***Autoimmune polyendocrinopathy syndrome type I*** *-* Mucocutaneous candidiasis, hypoparathyroidism, and Addison disease. POI affects up to 50% of females, rarely before menarche.	*AIRE* Common in Finland, Iranian Jewish community, Sardinia	AR
***Woodhouse-Sakati Syndrome*** *-* POI usually presenting as primary amenorrhea followed by diabetes, hypothyroidism, alopecia, extrapyramidal movements, sensorineural hearing loss.	*DCAF17* Strong founder effect in Saudi Arabia and Qatar	AR
***Perrault syndrome*** *-* Sensorineural deafness, short stature and POI, the majority with primary amenorrhea.	*HSD17B4, HARS2, LARS2 CLPP C10orf2, CLDN14+ SGO2, KIAA0391, ERAL1 account for 50% of cases*	AR
***Blepharophimosis, ptosis, epicanthus inversus syndrome (BPES)*** *-* Type 1 variant associated with POI, usually secondary amenorrhea. Narrow eyelid opening and ptosis may be evident at birth.	*FOXL2*	AD
***Galactosemia*** *-* POI (>80% of females) presents with primary (50%) or secondary amenorrhea (50%) after neonatal metabolic presentation.	*GALT*	AR
***Pseudohypoparathyroidism type 1a*** *-* Childhood onset hypocalcemia, high phosphate, cataracts, seizures. ~50% delayed puberty. POI caused by partial gonadotropin resistance.	*PHP1a*	AD
***Ovarioleucodystrophy*** - Early onset ataxia with white matter disease. POI with primary or secondary amenorrhea and ovarian atrophy.	*EIF2B2, EIF2B4*	AR
***Ataxia telangiectasia*** *-* Early onset ataxia <2 years followed by telangiectasia. POI; rare pregnancies recorded.	*ATM*	AR
***Premature aging syndromes*** *-* Scleroderma-like skin change from adolescence. POI usually as secondary amenorrhea. Diabetes mellitus in 80%. Rare pregnancies reported.	*WRN,ANTXR1*	AR
***Progressive external ophthalmoplegia*** *-* Primary amenorrhea in ~50%. Presentation with ophthalmoplegia and ptosis but rare POI with no other features.	*POLG *	AR
***Fanconi anaemia*** *-* POI usually presents at >20 years of age. Also associated with developmental delay, short stature, cardiac defects, genitourinary and gastrointestinal abnormalities, craniofacial abnormalities, VACTERL association, radial x-ray abnormalities.	*FANCA, FANCM, FANCL FANCD1/BRCA2, FANCU/XRCC2*	AR

**Table 2.** Selected examples of genes in which pathogenic variants have been associated with nonsyndromic primary ovarian insufficiency, grouped by main mechanism of action

Mechanism	Genes
Ovarian stimulation and function	*LHCGR* [37]*, FSHR* [38]*, GDF9* [39]*, BMP15* [40]*, FIGLA* [41]
Steroidogenic defect	*STAR* [42]*, CYP17A1* [43]*, CYP19A1* [43]
Ovarian development	*NR5A1* [44]*, FOXL2* [45]*, POLR3H* [46]
Meiosis and DNA repair	*MSH4* [47]*, MSH5* [48]*, HFM1* [49]*, BRCA2* [50]*, REC8* [33]*, SMC1B* [33]*, SYCE1* [51]*, CPEB1* [52]*, STAG3* [53]*, PSMC3IP* [54]*, DMC1* [55]*, MCM8* [56]*, MCM9* [57]*, NUP107* [58]*, CSB-PGBD3* [59]*, SPIDR* [60]*, TUBB8* [61]*, MEIOB* [62]*, ZSWIM7* [63]*, YTHDC2* [64]
Primary germ cell maintenance	*NOBOX* [65]*, SOHLH1* [66]

Some countries offer panel-based sequencing approaches to selected women (eg, via the National Genomics Test Directory in the United Kingdom), but, broadly, expanded genetic testing beyond karyotyping and fragile X screening is not currently recommended in clinical practice [17]. The diagnosis of POI remains unexplained in 50% to 80% of women, which can be frustrating and impedes an individualized approach to management.

## Case 3—46,XY Differences of Sexual Development

A girl was referred just after her 15th birthday because she had not started puberty. Her parents had also had late puberty so did not worry initially. On examination, there was no breast development (B1), minimal pubic hair (P2), no signs of genital or systemic virilization, and no other notable features such as hyperpigmentation. No inguinal gonads were palpable. Her height and weight were on the 25th percentile in keeping with delayed puberty (mid-parental height 50th-75th percentile). Her blood pressure was normal (110/65 mm Hg). Basal gonadotropins were elevated (LH 44 IU/L, FSH 77 IU/L) and electrolytes normal (sodium 140 mmol/L, potassium 4.1 mmol/L).

After discussion with the parents and young person, further tests were undertaken. The karyotype was 46,XY; a transabdominal ultrasound suggested a possible very small “müllerian structure” and intraabdominal gonad on the right; and adrenal investigations were normal (basal cortisol, basal adrenocorticotropin, cosyntropin stimulation test, urine steroid profile analysis). Basal tumor markers were negative (α-fetoprotein, β–human chorionic gonadotropin [β-hCG]). Abdominal magnetic resonance imaging (MRI) showed a small vestigial uterus and small bilateral intra-abdominal streak-like gonads. A likely diagnosis of complete gonadal dysgenesis was made (“Swyer syndrome”).

Further sharing of information was undertaken with specialist psychology input and multidisciplinary team discussion. It was felt that the patient’s gender identity was female and she wanted to start estrogen replacement for induction of puberty. After counseling, a clinical genetic gene panel for differences of sexual development (“DSD”) was performed that identified a de novo hemizygous missense variant in the HMG box of SRY. With informed consent, vaginal examination, vaginoscopy, and laparoscopy were performed under anesthetic. A typical clitoris, vagina, and cervix was seen, and small uterus. During the same procedure, bilateral streak-like gonads were removed, as had been discussed and consented to in advance. Cryopreservation of gametes was not felt to be an option. Histology showed a streak gonad on the left and a slightly larger severely dysgenetic gonad with well-contained gonadoblastoma on the right. Ongoing support was given and pubertal induction undertaken, which resulted in progressive uterine growth on ultrasound imaging 1 year later.

The mostly likely diagnosis in a 46,XY girl presenting with absent puberty and elevated gonadotropins is complete gonadal (testicular) dysgenesis (sometimes known as Swyer syndrome) [1, 68]. However, complete blocks in androgen synthesis such as Leydig cell hypoplasia (LHCG receptor) and complete combined 17α-hydroxylase/17,20-lyase deficiency (17αOHD) must not be overlooked, especially if LH is higher than FSH. 17αOHD is a rare form of congenital adrenal hyperplasia associated with hypertension and hypokalemia, so checking blood pressure and serum electrolytes at presentation in all 46,XY girls or women is important [69]. Measurements of progesterone, a urine steroid profile, and adrenocorticotropin-stimulation test should be performed if there is any clinical suspicion of 17αOHD or if the diagnosis is otherwise unclear, especially when testes are identified or when LH is dominant. The presence of virilization in a 46,XY girl (often with some breast development and mildly elevated gonadotropins) expands the differential diagnosis to include 17β-hydroxysteroid dehydrogenase deficiency type 3 (17βHSD), 5α-reductase deficiency type 2 (5αRD), defects in *NR5A1* (also known as SF-1), and very rarely partial gonadal dysgenesis and partial androgen insensitivity syndrome, although these latter conditions would usually be diagnosed earlier with atypical genitalia in infancy. Of note, women with complete androgen insensitivity syndrome usually have breast development and gonadotropins are not usually substantially elevated (especially FSH), so usually would not present with absent puberty with elevated gonadotropins (see Fig. 1). A full clinical examination and consideration of associated features is warranted given that up to one-fourth of infants with 46,XY DSD have an associated anomaly, and sometimes these individuals may first present during the teenage years with renal issues (eg, WT1/Frasier syndrome) [70]. Prior history is also important, such as primary adrenal insufficiency due to congenital lipoid adrenal hyperplasia (eg, steroidogenic acute regulator protein, CYP11A1), especially if a karyotype test was not conducted previously [71].

A transabdominal pelvic ultrasound is an essential initial investigation of 46,XY DSD and can help narrow a diagnosis. Imaging is very important to identify and characterize any gonads present and to define uterine structures, but is often not straightforward. Although transabdominal pelvic/inguinal ultrasound is a useful initial investigation, MRI can be more useful in delineating complex anatomy during the teenage years. Müllerian structures are usually present on imaging in complete gonadal dysgenesis but the uterus and upper vagina are absent in conditions affecting androgen synthesis (eg, 17αOHD, 17βHSD, 5αRD).

Importantly, 46,XY DSD conditions such as complete gonadal dysgenesis can be associated with a risk of gonadoblastoma and malignancy of up to 40%, depending on underlying diagnosis and age [72, 73]. A prophylactic gonadectomy should be performed in any child with a Y chromosome who has a streak or dysgenetic intraabdominal gonad [74]. Biomarkers (eg, α-fetoprotein, β-HCG) may suggest gonadoblastoma but are more reliable in the context of established germ cell tumors and ultimately a diagnosis can be made only on histopathological analysis after gonadectomy. Ascertaining the correct timing and approach to gonadectomy requires informed conversations with the child, family, and involved health care professionals [75, 76]. An additional consideration is deciding whether to attempt experimental cryopreservation of small gonads in selected young people. Cryopreservation will usually not be possible for 46,XY girls presenting with complete gonadal dysgenesis and elevated gonadotropins. Sometimes the appropriateness of cryopreservation needs to be decided on intraoperatively with prior consent in place. A new diagnosis of 46,XY DSD in children and adolescents requires that the need for age-appropriate, paced information sharing be balanced with the need to address medical issues with a degree of urgency [1].

Genetic testing in the setting of 46,XY DSD has become increasingly available with reasonable turnaround times in recent years in many countries [77]. Variants and copy number variants in many genes have been implicated in DSD pathogenesis (*SRY*, *NR5A1)* and the number of associated genes is increasing [78]. Overall, a diagnosis can be achieved for about 25% of individuals with complete gonadal (testicular) dysgenesis, but the percentage is much higher where there is clear evidence of a steroidogenic defect [79]. Identifying a pathogenic variant can cement the diagnosis and can yield important information for the wider family; for example, variants in *NR5A1* (*SF-1*) can be associated with a range of phenotypes within a pedigree, including POI in 46,XX girls and women [44]. Genetic testing, as always, needs to be approached with care and with appropriate counseling.

## Discussion

These 3 clinical vignettes outline 3 key differential diagnoses to consider when approaching the management of a young person with absent puberty and elevated gonadotropins. Taking a full history and performing a sensitive, systematic examination can help reach a diagnosis.

A karyotype test must be performed early on in the diagnostic process; the finding of a complete or partial X-chromosome deletion prompts full workup for TS, and the finding of any Y-chromosome material requires planning for a gonadectomy to manage tumor risk. Pelvic imaging and AMH measurement are useful when investigating 46,XY DSD, possibly helpful when considering fertility options in TS, and of no considerable benefit in POI. Other investigations may be appropriate depending on the likely diagnosis. Fig. 1 shows how the presentations of primary amenorrhea discussed in this review can be approached using a diagnostic algorithm. Several issues that arise in clinical practice are not reflected by this simplified diagram. For example, breast development is not often a clear binary characteristic; limited breast development may be seen in some girls with elevated gonadotropins, especially if they are overweight. Regarding uterine imaging, a uterus that has never been exposed to estrogen can be so small as to appear absent even on MRI scanning—a “clandestine uterus” [80, 81]. In the presence of estrogen deficiency, an absent uterus can be declared only after several months of estrogen administration and repeat imaging.

The clinical scenarios discussed here highlight several important management principles when planning the care of a girl presenting with absent puberty and hypergonadotropic gonadal insufficiency. We will now expand on some of these further.

## Pubertal Induction

Regardless of cause, young people presenting with delayed or arrested puberty require pubertal induction. Careful liaison with a pediatric endocrinologist is needed to maximize breast and uterine development using as physiological an approach as possible [82]. Studies of serum estradiol concentrations in puberty show that levels begin to rise from age 10 with the median age of menarche being 13 years. To mimic this physiology, induction of puberty ideally should take place at ages 11 to 12 years over 2 to 3 years with the dose of estradiol increasing from about 10% of the adult dose to a full physiological dose [17]. There are several pubertal induction regimens that vary by country [82-85]. A common approach in Europe is to gradually increase transdermal estrogen patches from one-fourth of a 25-mcg patch worn for 4 days of the week, to a full patch changed twice weekly [83]. This dose can be titrated against individual variable response, assessed either by Tanner staging or by transabdominal ultrasound of the uterus, and dose adjustments departing from the fixed regimen can be made accordingly [86]. The combined oral contraceptive pill is not recommended for use in pubertal induction: It contains supraphysiological doses of synthetic estrogen, which is associated with greater risks of hypertension, thrombosis, and dyslipidemia [87-89].

Progestogens should be commenced for uterine protection after at least 2 years of unopposed estrogen or once breakthrough bleeding occurs [17]. The timing of the introduction of progesterone can be further guided by ultrasound assessment of endometrial thickness. The average age of presentation of primary amenorrhea is usually older that age 14 and often as late as age 18 years. In such circumstances the dose of estrogen may need to be increased at a faster pace than usual, according to individual response and preferences and considering optimal psychological benefit [90]. Pubertal induction in TS can pose specific challenges, including balancing adequate estrogen replacement with optimizing growth, as discussed earlier [9]. All individuals on estrogen replacement should have regular follow-up consultations to review the goals of treatment, psychosexual well-being, and hypoestrogenic symptoms.

Once pubertal induction is complete, individuals with hypergonadotropic hypogonadism require ongoing hormone replacement with estrogen and progesterone at physiological doses (HRT) until approximately the age of natural menopause. Maintenance HRT needs to be tailored to the individual patient’s needs, preferences, and hypoestrogenic symptoms. The HRT regimen can use cyclical progesterone with withdrawal bleeds or continuous progesterone with no bleeds. Estrogen replacement can be administered transdermally, orally, or topically; estradiol esters and 17β-estradiol are most often used. There is no evidence that HRT confers an increased risk of breast cancer in women using lifelong estrogen replacement therapy up to the age of natural menopause [17]. Therefore, no change to standard breast cancer screening is required.

## Bone Health

Bone is an important target organ for estrogen, and estrogen deficiency is acknowledged to cause impaired bone mineral density (BMD) and an increased risk of fractures [91, 92]. BMD should be measured at diagnosis and at 5-year intervals throughout life [17, 93]. Supraphysiological doses of estrogen may be considered for those with low BMD who are younger than 50 years. Factors associated with a low BMD in the setting of ovarian insufficiency include low vitamin D, suboptimal calcium intake, primary amenorrhea, and low body mass index [94-96]. The importance of bone protection in the setting of ovarian insufficiency needs to be discussed from diagnosis: optimal dietary calcium and vitamin D intake, maintaining a healthy weight, weight-bearing exercise, and not smoking [97-99].

## Fertility Options

Fertility options will be limited for women with hypergonadotropic gonadal insufficiency but nevertheless a full discussion of the topic is important soon after diagnosis. This discussion should be revisited at intervals throughout follow-up, as the technology and opportunities in this field are evolving.

Assisted conception with donated oocytes has been used to achieve pregnancy for more than 20 years and remains the main fertility treatment option for the majority of women presenting with raised gonadotropins [100]. The availability of donated oocytes varies from country to country, and this option is not acceptable for some people. Adoption is also a commonly chosen route to family life. In the oncology setting, a small number of pregnancies have been achieved internationally using ovarian tissue cryopreservation approaches [101]. Despite possible discordance between gonadal type and gender identity, pregnancies from cryopreserved gonadal tissue in vitro maturation of sperm may be possible for individuals with 46,XY DSD in the future, the latter avoiding the potential risk of reintroducing tissue carrying a malignancy risk [75]. Even when follicular apparatus is found, fertility preservation is not usually possible for women with POI who have increased FSH. However, cascade genetic screening of family members of women with POI may allow presymptomatic intervention for women at risk of POI who have yet to develop it. Young women with mosaic TS and persistent ovarian function are potential candidates for oocyte cryopreservation; however, in the main, oocyte donation is the main fertility option for women with TS [9,102]. Pregnancies in TS need to be carefully monitored by an expert multidisciplinary team given the increased morbidity and mortality risks conferred by pregnancy in these women (aortic dissection, hypertension) [9, 103].

## Sexual Function

In many countries a substantial proportion of girls have sexual intercourse before age 18. This topic has to be approached cautiously but not neglected. If possible, an adolescent gynecologist should be available early in the young person’s journey so they can then be a familiar advisor when required. Several endocrine aspects are relevant in this area. For example, in postpubertal girls who have reached sexual maturity, vaginal estrogen may be required for adequate vaginal lubrication and testosterone may be added later if libido is low [104].

The chances of spontaneous fertility should also be discussed, even with those presenting with primary amenorrhea: Spontaneous conceptions do occur in POI and TS in approximately 1% to 5% of adult women with these conditions [2, 3, 5, 105-107]. There are no corresponding data for adolescents, although their chances of conception are likely to be much lower. It is important to address the possibility of pregnancy, however remote, as contraception is required for those not desiring to conceive.

## Psychological Considerations

Diagnoses associated with hypergonadotropic hypogonadism can be very difficult life experiences both for the young person and their parents, and signposting reliable information sources is essential from the outset.

A TS diagnosis in a young person is associated with specific psychological issues, including an increased risk of anxiety, social isolation, reduced self-esteem, and shyness. These problems can manifest in school and, later, in the workplace. The support of a clinical psychologist can help address these issues. Most women with TS have IQs in or above the normal range; 10% have learning difficulties, especially with the 45,X/46,X,r(X) karyotype, and neuropsychological review at key transition points during schooling is recommended. Timely pubertal induction and management of any hearing impairment can promote psychosocial well-being. Support groups can be very useful (eg, the Turner Syndrome Support Society UK).

A diagnosis of POI is challenging psychologically, and in adults has been associated with increased risk of depression, low levels of self-esteem, and negative effects on sexuality [108, 109]. It is a particularly difficult diagnosis in a young person, and access to a clinical psychologist and support groups (eg, The Daisy Network [UK]) is helpful. Regular specialist follow-up is recommended so young people have access to accurate and up-to-date fertility information and so the need for psychology support can be reassessed at intervals. Often crises arise some years after the original diagnosis, for instance, when a near relative achieves a pregnancy.

Early specialist psychological input from the time of presentation is also essential for a girl with 46,XY DSD. These young people often have delayed puberty and may be more immature than their peers, requiring graded information-sharing balanced with the need to progress pressing medical issues, as discussed earlier. Open dialogue is paramount and the young person needs be involved with decision-making processes and feel connected with the team; often, children can give appropriate consent or assent depending on the legal framework of the jurisdiction. Repeated assessment of gender identity is essential, especially when considering induction of puberty and other endocrine interventions. Consideration of different cultural backgrounds and preferences is also important. A DSD diagnosis can be challenging for parents and caregivers too, who have a lot to take in within a short space of time. Again, in addition to clinical psychology, there are resources for information sharing and support groups available (eg, dsdfamilies, www.dsdteens.org).

## Conclusion

A range of diagnoses, including TS, POI, and 46,XY DSD, may explain the presentation of delayed puberty and increased gonadotropins in a young girl. A judicious investigation plan, beginning with a karyotype test, facilitates a timely diagnosis. These are rare conditions and an awareness of relevant international consensus guidelines and research developments are useful when planning and directing clinical care. Multidisciplinary input, open communication, shared decision-making, and dedicated support groups can help young people and their families.

## Data Availability

No original data are included in this manuscript.

## Abbreviations

β-hCG: β–human chorionic gonadotropin
AMH: antimüllerian hormone
BMD: bone mineral density
DSD: differences/disorders of sex development
FSH: follicle-stimulating hormone
HRT: hormone replacement therapy
LH: luteinizing hormone
MRI: magnetic resonance imaging
POI: primary ovarian insufficiency
TPO: thyroid peroxidase
TS: Turner syndrome
